# A comparative study between effect of combined intravenous and nebulized amikacin versus intravenous amikacin alone in mechanically ventilated patients with ventilator-associated pneumonia (VAP)

**DOI:** 10.1186/s42077-020-00098-3

**Published:** 2020-10-07

**Authors:** Dalia M. El Fawy, Azza Yousef Ibrahim, Ahmed Mostafa Mohamed Abdulmageed, Eman Abo Bakr El Seddek

**Affiliations:** Department of Anesthesiology, Intensive Care and Pain Management, Faculty of Medicine, Ain-Shams University, Fifth settlement, Cairo, 11865 Egypt

**Keywords:** Amikacin, Nebulized, VAP, Ventilation, Intravenous, Antibiotics

## Abstract

**Background:**

Aerosolized antibiotic administration offers the theoretical advantages of achieving high drug concentrations at the infection site together with lower systemic absorption. This study aims to compare the effect of combining nebulized amikacin with intravenous amikacin to the effect of the usual intravenous route alone in the treatment of patients with ventilator-associated pneumonia and its impact on the duration of mechanical ventilation, laboratory, and clinical picture of the patients.

**Results:**

This study was carried out on 64 mechanically ventilated patients with Gram-negative VAP. The patients were divided into 2 groups. Group A included 32 patients treated with nebulized amikacin plus IV amikacin, and group B included 32 patients treated with IV amikacin alone. The duration of treatment for both groups was 8 days with a daily assessment of Clinical Pulmonary Infection Score (CPIS) and monitoring of clinical and laboratory parameters. Sputum cultures were obtained thereafter. In our study, the CPIS score and overall ICU mortality were less in the nebulized than in the IV group but the difference failed to be statistically significant. Increase of oxygenation level (Pao2/Fio2 ratio), organism clearance, decrease in serum creatinine level, duration of mechanical ventilation, and length of ICU stay were significantly different in favor of group A than group B.

**Conclusion:**

Nebulized and IV amikacin offered better oxygenation, organism clearance, less nephrotoxicity, and less duration of mechanical ventilation and ICU stay than the IV group. Combined and IV routes were comparable regarding the decrease in CPIS score and ICU mortality with no significant difference between them. However, we prefer to use the combined regimen for the mentioned reasons. Further large-scale studies are required to confirm these findings and to establish a definite conclusion.

## Background

Ventilator-associated pneumonia (VAP) is a term defined as an infection of the lower respiratory tract that is associated by endotracheal intubation which can cause significant morbidity and mortality in the intensive care unit (ICU). VAP is one of the most common healthcare-associated infections arising in the ICU (Vincent et al., 1995). About 10% of mechanically ventilated patients will develop VAP, with its risk rising with the increased duration of mechanical ventilation, reaching a peak incidence on the fifth day after intubation (Cook et al., 1998). Further, VAP is associated with a significant morbidity as it increases the length of stay in the ICU, duration of mechanical ventilation, and hospital stay (Safdar et al., 2005).

VAP caused by multidrug-resistant organisms (MDROs) is associated with much more mortality (Bercault & Boulain, 2001; Vallés et al., 2007). Many potential multidrug resisting organisms include *Acinetobacter* spp., *Klebsiella* producing carbapenemase, ESBL producing enterobacteriacea, *Pseudomonas aeruginosa*, *Stenotrophomonas maltophilia*, and methicillin-resistant *Staphylococcus aureus*.

Combined assessment of clinical data, microbiological results, and radiological findings is needed to diagnose VAP. There are no simple and easy methods to diagnose VAP. Whenever there is a high clinical suspicion of VAP, empirical antimicrobial must be administered immediately as both delayed management and inadequate treatment are accompanied by a higher morbidity and mortality incidence (Chastre & Fagon, 2002).

The current guidelines recommend coverage of Gram-negative bacilli (GNB) empirically with a carbapenem, piperacillin-tazobactam, and a third- or fourth-generation cephalosporin, in combination with an aminoglycoside or a fluoroquinolone (American Thoracic Society and Infectious Diseases Society of America, 2005). One of the sequelae of a greater prevalence of resistance to antimicrobials is an increased incidence of inadequate treatment of infection. Few alternatives are available for the treatment of Gram-negative multidrug-resistant (MDR) bacilli. Administration of inhaled antibiotics offers the delivery of high drug levels in the lung tissue together with reduction of the systemic toxicity that associates intravenous (IV) antibiotics. The concentration of the inhaled antibiotics in the respiratory secretion may reach 20-to 100-folds higher than in vitro minimum inhibitory concentration (MIC) of the organisms being treated (Palmer, 2009).

A higher resistance to antimicrobials together with the shortage of development of new antimicrobial necessitates novel treatment strategies for optimization of the pharmacodynamics of the already existing antimicrobials. This may aid in preserving antibiotic efficacy, decrease emergence of resistance, and provide a pharmaco-economic benefit (Jaruratanasirikul & Sriwiriyajan, 2003).

## Methods

After approval of the research ethical committee and obtaining written informed consent from patients’ legal guardians, this randomized observational prospective study was conducted on sixty-four patients (male and female) scheduled with ventilator-associated pneumonia (VAP) which were included in this study. They were randomly allocated into two equal groups using computer-generated randomized table and sealed opaque envelopes. The groups were group A (*n* = 32), which received combined nebulized and intravenous amikacin, and group B (*n* = 32) which received intravenous amikacin alone.

The study included patients with either sex with age older than 18 years who were admitted to the Multidisciplinary Intensive Care Unit. VAP was defined as any pneumonia that occurs 48–72 h or thereafter following endotracheal intubation, characterized by the presence of a new or progressive infiltrate, signs of systemic infection (fever, altered white blood cell count), changes in sputum characteristics, and detection of a causative agent.

Patients whose legal guardians refused to participate in the study, those who had known allergy or bacterial resistance to amikacin, and those who had creatinine clearance less than 60 mL/h or PaO2/PiO2 less or equal to 100 mmHg were excluded from the study. If any of the patients experienced increasing Clinical Pulmonary Infection Score (CPIS) or with positive cultures after 8 days, he/she was omitted from the study and shifted to intravenous amikacin 20 mg/kg/day.

### Study procedures

After written informed consent was obtained from legal guardians, patients were randomly assigned to receive either combined intravenous and nebulized amikacin or intravenous amikacin alone after eligibility criteria have been met in both groups. VAP was defined as any pneumonia that occurs 48–72 h or thereafter following endotracheal intubation, which is characterized by the presence of any new or progressive infiltrate, together with signs of systemic infection (such as fever, altered white blood cell count), changes in sputum characteristics, and detection of the causative agent. Nebulization of amikacin 10 mg/kg (in 10 mL) in a single daily dose together with a single dose of 10 mg/kg/day of intravenous amikacin was performed in group A while nebulization of 10 mL of normal saline together with 20 mg/kg/day in a single daily intravenous dose was performed in group B; both groups received standard ICU treatment protocol of thromboprophylaxis, antistress medications, and antipyretics. Daily assessment of Clinical Pulmonary Infection Score (CPIS) and monitoring of clinical and laboratory parameters were performed and recorded. End of study was authorized for patients with increasing CPIS or with positive cultures after 8 days and will be shifted to intravenous amikacin 20 mg/kg/day. Nebulization was performed with vibrating plate nebulizers using specific ventilator settings. The primary outcome of the study was to compare the mechanical ventilatory days between study groups, while secondary outcomes were to assess the length of ICU stay, nephrotoxicity, oxygenation parameters, clinical improvement, and ICU mortality rate.

(1) Cure of VAP was defined as successful weaning, reduction of both clinical and biological signs of infection, a decrease in CPIS below 6, and lower respiratory tract specimens either negative or with non-significant. (2) Persisting VAP is defined as a lack of improvement of clinical and biological signs, CPIS greater than 6, failure of weaning, and significant concentrations of the causative organism that still persists in the lower respiratory tract specimens. (3) Recurrence of VAP is defined as initial cure after 8 days of antimicrobial therapy which is followed by post-treatment relapse of VAP. (4) Superinfection is defined as initial cure after 8 days of antimicrobial therapy followed by post-treatment relapse of VAP caused by any pathogens other than the initial causative organism. CPIS parameters are as follows (Table 1).

**Table 1:** CPIS score parameters (Schurink et al., 2004)

**1. Body temperature** ≥ 36.5 or ≤ 38.4 = 0 point ≥ 38.5 or ≤ 38.9 = 1 point ≥ 39 or < 36.5 = 2 points **2. Total leucocytic count** ≥ 4000 or ≤ 11.000 = 0 point < 4000 or > 11.000 = 1 point Rod form ≥ 50% = add 1 point **3. Tracheal secretion** Tracheal secretion (−) = 0 point Tracheal secretion with less purulence = 1 point Abundant purulent secretion = 2 points **4. Oxygenization** Pa02/Fi02, mmHg >240 or ARDS (ARDS: Pa02/Fi02 < 200, Pa02/Fi02 < 200, PAWP ≤ 18 mmHg and bilateral acute infiltration) = 0 point Pa02/Fi02, mmHg ≤ 240 or ARDS = 2 points	**5. Pulmonary infiltration in chest X-ray** No infiltration = 0 point Diffuse infiltration = 1 point Localized infiltration = 1 point **6. Progression in pulmonary infiltration** Radiographic progression (−) = 0 point Radiographic progression (+) (after the exclusion of HF and ARDS) = 2 points **7. Pathogenic bacteria in tracheal aspirate culture** No or few pathogenic bacteria = 0 point Moderate or high levels of pathogenic bacteria = 1 point Pathogenic bacteria to be seen in Gram staining, add 1 point

### Sample size

Using PASS program setting, the alpha error is 5% and power is 80%. Result from a previous study (Qi et al., 2016) showed that 70% of cases were cured with nebulized amikacin and ceftazidime compared to 55% cured by the intravenous form of these drugs. Based on this, the needed sample is 32 cases per group (total 64).

### Statistical analysis

The collected data will be revised, coded, tabulated, and introduced to a PC using Statistical Package for Social Science (SPSS 15.0.1 for windows; SPSS Inc, Chicago, IL, 2001). Data will be presented as mean and standard deviation (± SD) for quantitative parametric data, and median and interquartile range for quantitative nonparametric data. Frequency and percentage will be used for presenting qualitative data. The suitable analysis will be done according to the type of data obtained. The Student *T* test or Mann-Whitney test will be used to analyze quantitative data, while the chi-square test and Fisher extract test will be used to analyze qualitative data. *p* value < 0.05 will be considered statistically significant.

## Results

**Fig. 1:**
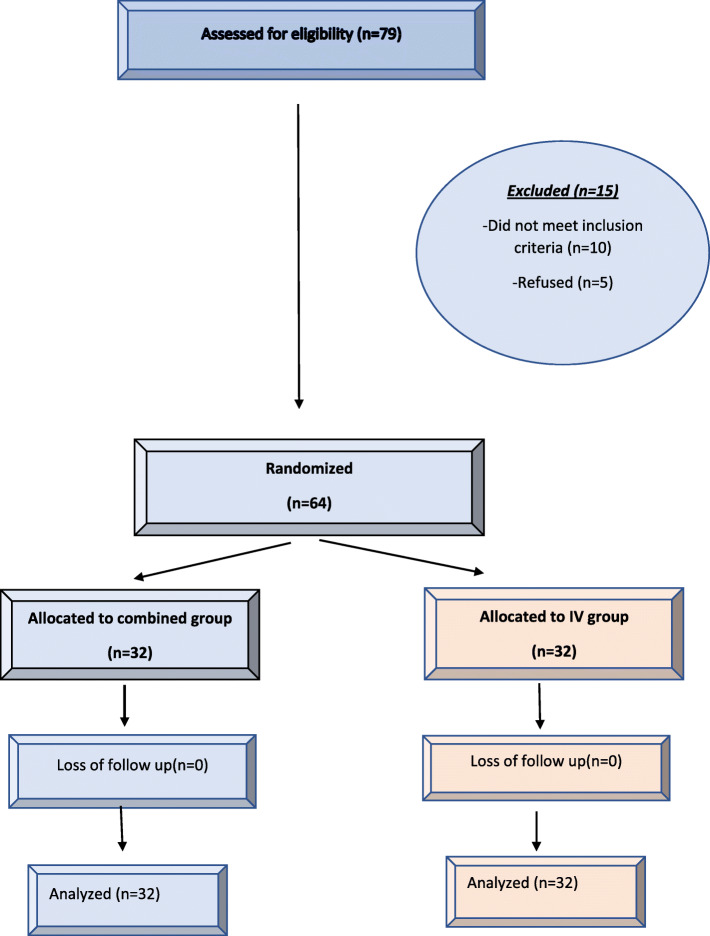
Patient flow chart

Seventy-nine patients were screened for this study: 10 patients did not meet inclusion criteria and 5 patients’ legal guardians refused to participate in our study. The remaining 64 patients were randomly allocated into the groups of the study (Fig. 1).

### Patient data

There were no significant differences in patients between the groups as regards age, sex, and causes of ICU admission as shown in Table 2.

**Table 2:** Comparison between study groups as regards age, sex, and causes of ICU admission

		Total (***N*** = 64)	Group A (***N*** = 32)	Group B (***N*** = 32)	A/B
Age (years)	Mean ± SD	59.3 ± 17	58.0 ± 14.4	59.3 ± 17.4	0.791^^^
	Range	28.0–88.0	25.0–90.0	28.0–88.0	
Sex	Male	49 (76%)	21 (65.0%)	28 (87%)	0.288^#^
	Female	15 (24%)	11 (35.0%)	4 (12%)	
Comorbidities	DM	17 (26%)	8 (25.0%)	9 (28%)	0.723^#^
	HTN	20 (31%)	10 (30.0%)	10 (30%)	0.736^#^
Causes of admission	Resp.	52 (81%)	30 (95.0%)	22 (69%)	0.151^#^
	Neurological	26 (40%)	11 (35.0%)	15 (47 %)	0.519^#^

^^^Independent *t* test

^#^Chi-square tests

### Causative organisms

*Pseudomonas* infection was(60% vs. 35%, *Klebsiella* infection was 35% vs. 20%, and *Acinetobacter* infection was 10% vs. 35% in group A vs. B. There were no significant differences regarding organisms revealed from cultures as shown in Table 3.

**Table 3:** Comparison between study groups as regards organisms revealed from cultures before treatment

	Group A	Group B	A/B
*Klebsiella*	11 (35.0%)	6 (20.0%)	0.288
*Acinetobacter*	3 (10.0%)	11(35.0%)	0.058
*Pseudomonas*	19 (60.0%)	11(35.0%)	0.113
*Citrobacter*	0 (0.0%)	1 (5.0%)	0.311
*Staphylocci*	0 (0.0%)	2 (10.0%)	0.147
*Provedinetia*	1 (5.0%)	1 (5.0%)	1.000
*Proteus*	1 (5.0%)	1 (5.0%)	1.000
*Enterobacter*	1 (5.0%)	0 (0.0%)	0.311

Data are presented as number of cases and percentage of each to total number of cases in each group. *p* value > 0.05 NS

*x*^*2*^ chi-square test

### CPIS score and clinical cure

In group A, the CPIS score was ≤ 6 in 75.0%, while in group B, it was ≤ 6 in 55% without significant differences as shown in Table 4.

**Table 4:** Comparison between study groups as regards the CPIS score after treatment (clinical cure)

CPIS	Group A	Group B	A/B^#^
**≤ 6**	24 (75.0%)	18 (55.0%)	0.185
**> 6**	8 (25.0%)	14 (45.0%)	

^#^Chi-square test

### Treatment efficiency, length of ICU stay, and adverse effects

There was a significant improvement of oxygenation before and after treatment within group A (*p* 0.006), while group B shows no significant differences (*p* 0.212) (

**Table 5:** Comparison between case and control groups as regards oxygenation (Pao2/Fio2)

		Group A	Group B	A/B^**^**^
**Before**	**Median (IQR)**	164.5 (147.8–229.5)	164.0 (147.0–194.3)	0.570
	**Range**	105.0–400.0	103.0–255.0	
**After**	**Median (IQR)**	191.0 (162.8–230.3)	177.5 (150.0–221.3)	0.279
	**Range**	142.0–402.0	120.0–285.0	
**Difference**	**Median (IQR)**	20.5 (− 6.8–41.5)	24.0 (− 10.0–54.8)	0.797
	**Range**	− 30.0–60.0	− 100.0–150.0	
***p***^**#**^		0.006*	0.212	

Negative values indicate reduction

*Md* Median, *IQR* interquartile range

^**^**^Mann-Whitney test

^**#**^Wilcoxon signed rank test

*Significant *p* value > 0.05 NS; *p value < 0.05 S; **p value < 0.001 H

Table 5).

LOS was 21.5 vs. 25.5 in group A vs. B respectively with a significant reduction in group A versus group B (*p* 0.037). The duration of MV was 19 vs. 23 in group A vs. B, respectively, with a significant reduction (*p* 0.045) of ventilator days a shown in Table 6.

**Table 6:** Comparison between case and control groups as regards the duration of MV and length of stay (days)

		Group A	Group B	A/B^**^**^
**Duration MV**	**Median (IQR)**	19.0 (16.0–26.3)	23.5 (21.3–26.0)	0.037*
	**Range**	13.0–90.0	20.0–38.0	
**LOS**	**Median (IQR)**	21.5 (16.3–27.0)	25.5 (23.0–28.5)	0.045*
	**Range**	15.0–90.0	20.0–38.0	

*IQR* interquartile range

**^**sign is to indicate the use of mann whitney test

*statistical significance of the value according to the mentioned test

There was significant rising of creatinine level in group B after treatment (*p* < 0.001), while there was no significant rise in creatinine level in group A after treatment. Also, there was a significant difference between groups A and B (*p* 0.003) after the end of treatment (Table 7).

**Table 7:** Comparison between case and control groups as regards creatinine (mg/dL)

Creatinine		Group A	Group B	A/B^**^**^
**Before**	**Median (IQR)**	1.10 (0.83–1.30)	1.15 (0.93–1.30)	0.594
	**Range**	0.60–2.30	0.80–1.80	
**After**	**Median (IQR)**	1.00 (0.73–1.28)	1.30 (1.20–1.50)	0.003*
	**Range**	0.20–2.00	0.80–2.50	
**Difference**	**Median (IQR)**	0.00 (− 0.28–0.10)	0.10 (0.03–0.28)	0.013*
	**Range**	− 2.10–1.00	0.00–1.60	
***p***^**#**^		0.505	< 0.001*	

Negative values indicate reduction

*Md* median, *IQR* interquartile range

^**^**^Mann-Whitney test

^**#**^Wilcoxon signed rank test

*Significant

### Microbiological response

The clearance of organism was 84.4% vs. 25%, resistance was 6.3% vs. 28.1%, and superinfection was 3.1% vs. 21.9%, while combined resistance and superinfection was 6.3% vs. 25% in group A vs. B, respectively. There was a significant difference between groups A and B as regards organism clearance after treatment. This is shown in Table 8.

**Table 8:** Comparison between study groups as regards organism clearance after treatment

	Group A	Group B	***x***^**2**^	***p*** value
**No growth**	27 (84.4%)	8 (25.0%)	20.43	< 0.001**
**Resistance**	2 (6.3%)	9 (28.1%)	4.952	0.036*
**Superinfection**	1 (3.1%)	7 (21.9%)	3.571	0048*
**Resistance and superinfection**	2 (6.3%)	8 (25.0%)	3.721	0.045*

Chi-square test

*Significant

**Highly significant

### ICU mortality

The mortality was 19 (60%) in group A vs. 26 (80%) in group B. The mortality was higher in group B than in group A but without achieving a statistically significant difference (Table 9).

**Table 9:** Comparison between study groups as regards ICU mortality

Time	Group A	Group B	A/B^#^
**Death**	19 (60.0%)	26 (80.0%)	0.168

^#^Chi-square test

## Discussion

Endotracheal tube placement elevates the risk of developing VAP by 6–20-fold when compared to critically ill patients who are not intubated, with attributable mortality 47% in patients with VAP patients versus 22% in the total ICU population (Niederman et al., 2005).

Intravenous antibiotics do not reach a bactericidal concentration in all different tissues of lungs and intravenously administered antibiotics are primarily detected in respiratory segments of lungs, but not in sputum, so increasing the daily dosage and combining different IV antibiotics render patients to more of their side effects. Nebulized antibiotic administration offers the theoretical advantages of achieving high drug concentrations at the infection site together with low systemic absorption, thereby decreasing their side effects. Nebulized antibiotics have been shown to be useful adjuncts to systemic antibiotic therapy for reducing morbidity and mortality caused by VAP (Dhand, 2007).

The findings of the present study were that VAP treatment with nebulized amikacin was associated with less ventilator and ICU days, higher oxygenation (PaO2/FiO2) rates, higher bacterial clearance with less resistance and superinfection, and less nephrotoxicity when used as adjunctive therapies for the treatment of VAP caused by MDR Gram-negative bacteria. There were less VAP-related mortality and a higher CPIS score in the combined group compared to the IV group, but the difference was not statistically significant.

In the study by Lu et al., forty patients with VAP that is caused only by *Pseudomonas aeruginosa* were included in a randomized comparative trial, comparing nebulized ceftazidime plus nebulized amikacin versus IV ceftazidime plus amikacin. Twenty patients received nebulized ceftazidime (15 mg/kg/3 h) and amikacin (25 mg/kg/day) versus seventeen patients receiving intravenous ceftazidime (90 mg/kg/day, continuous administration) and amikacin (15 mg/kg/day). They found that the clearance of organism was 70% vs. 55% (*p* 0.33) and resistance was 15% vs. 30% (*p* 0.26), while superinfection was 15% vs. 15% in nebulized versus IV groups, respectively, without statistically significant difference (Lu et al., 2011).

Although Lu et al. found a higher clearance rate in the nebulized group compared to the IV group, the lack of a statistical significance may be attributed to the smaller sample size (forty patients), using a different antibiotic (ceftazidime) which has different pharmacodynamics and pharmacokinetics on nebulization, using nebulized antiobiotics for 8 days without IV adjunct as we used in our study, and finally the use of vibrating mesh nebulizer instead of jet nebulizer in our study. Jet nebulizers are able to deliver smaller antibiotic particles deeper into the bronchial tree.

Diamantis et al. did a retrospective study in which forty-three patients with VAP received a combination of nebulized and intravenous antibiotics compared to intravenous antibiotics alone but the used antibiotic was colistin. The first group received aerosolized (1 million unit every 12 h) plus IV colistin (3 million every 8 h) versus 43 control patients who had received IV colistin (3 million every 8 h) alone. Clearance of organisms was 50% in the nebulized group versus 45% in the control group without statistically significant differences (*p* 0.67), which may also be attributed to the smaller sample size and different colistin nebulization pharmacokinetics (Kofteridis et al., 2010).

Lu and Luo studied 165 patients with VAP caused by *P*. *aeruginosa* and *A*. *baumannii*. The sensitive strain group included 122 patients with VAP caused by *P*. *aeruginosa* and *A*. *baumannii* susceptible to β-lactams, aminoglycosides, or quinolones and treated with intravenous antibiotics for 14 days. The multidrug-resistant strain group included 43 patients with VAP caused by multidrug-resistant *P*. *aeruginosa* and *A*. *baumannii* and treated with high-dose nebulized colistin (5 million units every 8 h) either in monotherapy (*n* = 28) or combined to 3-day intravenous aminoglycosides for 7–19 days. They found that the median duration of MV was 18 days in the nebulized group while in the control group it was 38 days with a very high statistically significant differences (*p* 0.001). On the other hand, the median of LOS in the nebulized group was 25 days, while in the control group, it was 54 days with significant differences (*p* 0.001). This similarity to our results may be attributed to the large sample size, the combined use of nebulization and IV routes, and the use of jet nebulizers (Lu et al., 2012).

Ammar and Abdalla studied ninety patients with VAP pneumonia. Group A received IV amikacin 20 mg/kg/day and meropenem 2g/8 h. Group B is the same as group A together with nebulized amikacin 25 mg/kg/day. Group C received IV amikacin 20 mg/kg/day, nebulized amikacin 25 mg/kg/day, and extended infusion of meropenem 2 g/kg/8 h over 3 h. Group B showed a highly statistically significant reduction in ventilator days 5.31 ± 1.86 vs 7.3 ± 2.1 days (*p* < 0.001) while group C also showed significant fewer ventilator days compared to group A, 4.22±1.32 vs 5.32 ± 1.86 (*p* < 0.011). Also, this similarity may be attributed to the larger sample size, combined use of nebulized and IV routes, and the use of jet nebulizers (Ammar & Abdalla, 2018).

In agreement with our study, Hassan et al. studied 133 patients in postcardiac surgery ICU. Inhaled amikacin was administered at a dose of 400 mg twice daily for 7 days to the nebulizer group whereas IV amikacin was administered in a dose of 20 mg/kg IV to the IV group for 7 days where both groups received IV piperacillin/tazobactam empirically according to the unit antibiogram. They found that there was a highly significant reduction in creatinine clearance values before and after the end of treatment between both groups with a rate of reduction of 10 mL/min (0–27) in the nebulized group vs 16 mL/min (8–30) in the IV group (*p* < 0.001) (Hassan et al., 2018).

The outcome and mortality were comparable in our study between both groups with no significant differences although it was less in the nebulized group (40% vs 60%, *p* 0.168). This is in agreement with all the previous studies mentioned. In Lu et al., the mortality rate in the nebulized group was 10% versus 5% in the IV group, without significant differences (*p* 0.55) (Lu et al., 2012). In the study by Diamantis et al., the VAP-related mortality was 16% in the nebulized group versus 26% in the control group without significant differences (*p* 0.289) (Kofteridis et al., 2010). In Ammar and Abdalla, the VAP-related mortality was 8% in the IV group (*p* 0.347) versus 5% in the IV and nebulized group (*p* 0.197) versus 4% in the nebulized and extended IV infusion group (*p* 0.717) with no significant differences (Ammar & Abdalla, 2018).

### Limitations

The first limitation to our study was that it was conducted in a single center, and the second limitation was that the treating intensivists were not blinded to the study arms.

## Conclusion

Nebulized amikacin is safe and effective in the treatment of VAP, although larger scale studies are needed to evaluate the efficacy and safety of nebulized antibiotics in the treatment of VAP and the possibility of their use as prophylactic and empirical stand-alone therapies, thus minimizing systemic antibiotic side effects and toxicity and to conclude whether the nebulization regimen is significantly superior to the IV alone regimen in treating ventilator-associated pneumonia.

## Data Availability

The datasets used and/or analyzed during the current study are available from the corresponding author on reasonable request.

## Abbreviations

A. baumannii: Acinetobacter baumannii
ARDS: Adult respiratory distress syndrome
DM: Diabetes mellitus
CHF: Congestive heart failure
CPIS: Clinical Pulmonary Infection Score
FiO2: Fraction of inspired oxygen
GNB: Gram-negative bacilli
HTN: Hypertension
ICU: Intensive care unit
IQR: Interquartile range
IV: Intravenous
LOS: Length of stay
MDR: Multidrug resistant
MV: Mechanical ventilation
PaO2: Partial pressure of oxygen in arterial blood
*P*. *aeruginosa*: *Psudomonas aeruginosa*
Resp: Respirator
VAP: Ventilator-associated pneumonia
